# Location-Aware Incentive Mechanism for Traffic Offloading in Heterogeneous Networks: A Stackelberg Game Approach

**DOI:** 10.3390/e20040302

**Published:** 2018-04-11

**Authors:** Kailing Yao, Yunpeng Luo, Yang Yang, Xin Liu, Yuli Zhang, Changhua Yao

**Affiliations:** 1 College of Communications Engineering, Army Engineering University, Nanjing 210007, China; 2 College of Information Science and Engineering, Guilin University of Technology, Guangxi 541000, China

**Keywords:** heterogeneous networks, location-aware, Stackelberg game, incentive mechanism

## Abstract

This article investigates the traffic offloading problem in the heterogeneous network. The location of small cells is considered as an important factor in two aspects: the amount of resources they share for offloaded macrocell users and the performance enhancement they bring after offloading. A location-aware incentive mechanism is therefore designed to incentivize small cells to serve macrocell users. Instead of taking the amount of resources shared as the basis of the reward division, the performance promotion brought to the macro network is taken. Meanwhile, in order to ensure the superiority of small cell users, the significance of them weighs heavier than macrocell users instead of being treated equally. The offloading problem is formulated as a Stackelberg game where the macro cell base station is the leader and small cells are followers. The Stackelberg equilibrium of the game is proved to be existing and unique. It is also proved to be the optimum of the proposed problem. Simulation and numerical results verify the effectiveness of the proposed method.

## 1. Introduction

One of the main advantages of deploying small cells (SCs) in heterogeneous networks (HetNets) is that they can help traffic offloading and provide better service for macrocell users (MUES) [1,2,3]. However, since SCs have dedicated small cell users (SUEs) to serve, they will not be helpful willingly and the macrocell base station (MBS) has to provide some reward to incentivize them [4]. The location of SCs has a great impact on the traffic offloading process. On one hand, it decides whether a MUE can be or should be offloaded. On the other hand, it affects the contribution each SC makes for the macro network after offloading. Therefore, this article focuses on designing a location-aware incentive mechanism to solve the traffic offloading problem in the HetNet.

In many existing works where the incentive mechanism in the HetNet [5,6,7,8,9,10] were investigated, SCs divided the rewards given by the MBS according to the resource they shared. Such rules are not reasonable considering the heterogeneous locations of SCs. For example, if two SCs, one close to the MBS and the other far from the MBS, share the same amount of resources, they will obtain the same amount of reward under the existing rules. However, since MUEs in the former SC can already receive good service before offloading, the performance promotion brought by the former SC will be less than the latter one. That is to say, it makes less contribution and it is reasonable to obtain less rewards. Therefore, the traditional reward division approach seems like it can be improved.

In order to make the incentive mechanism more reasonable and effective, a new reward division method which is based on the individual contribution each SC makes is proposed. The contribution is defined as the performance promotion each SC brings for the macro network after offloading. Instead of taking the more resources, the more rewards as the rule, in this article, the more promotion one SC contributes, the more rewards it obtains. Such a method makes SCs more rational, since if one cannot bring any promotion, it will not share any resources. By comparison, in traditional methods, in order to obtain more rewards, it will share many resources to occupy a large proportion of the total sharing resources.

The Stackelberg game has been widely used to solve problems in wireless networks where entities have different superiority [11,12,13,14,15,16,17,18,19,20,21,22,23]. In this article, we formulate the traffic offloading problem as a Stackelberg game, where the MBS is the leader and SCs are followers. The utility of each SC is the sum of two parts that relatively represents the reward from its dedicated SUEs and the MBS. Different from most existing work, in order to ensure the superiority of SUEs, we make the rewards from them weigh heavier than that from the MBS. This also helps SCs become more rational since they will take their dedicated SUEs more seriously when they make the resource sharing decision. In order to find the Stackelberg equilibrium (SE) of the game, we use backward induction to find the Nash equilibrium (NE) of the lower subgame first and the best strategy of the MBS subsequently. The SE is proved to be existing and unique and is proved to be the optimum of the proposed problem as well.

The main contribution of this article can be summarized as follows:

- The traffic offloading problem in a HetNet is formulated as a Stackelberg game, where the MBS is the leader and SCs are followers. The existence and uniqueness of the SE is proved. It is also proved that the SE is the optimum of the problem.
- The location of SCs is highlighted as an important factor which not only determines which MUE can be or should be offloaded, but also affects the performance promotion each SC makes if they serve MUEs. Different located SCs will contribute to different promotion even if they share the same amount of resources.
- A new reward division method that is based on the performance promotion each SC brings is proposed. Different from most existing works, the one who shares the most resources may not obtain the most rewards. This makes SCs more rational when they make the resource sharing decision.
- In order to ensure the superiority of dedicated SUEs, the reward each SC obtains from SUEs weighs more than that from the MBS. This helps SCs consider SUEs more during the resource sharing process rather than sharing many resources to compete for the reward from the MBS.

The rest of the paper is organized as follows. The related work is presented in Section 2. The system model and problem formulation are given in Section 3. The Stackelberg game is formulated and analyzed in Section 4. Simulation and numerical results are presented in Section 5 and a conclusion is made in Section 6.

## 2. Related Work

Offloading is an effective method that has been widely used to alleviate the burden of the overloaded network or application [24,25,26,27,28,29,30,31,32]. The authors of [25,26,27,28,29] investigated the data offloading problem in mobile systems while many other researchers focused on the application in HetNets [30,31,32]. However, since SCs will not help offloading willingly as it may degrade the service of their own users, the incentive mechanism should be designed to stimulate them.

The incentive mechanism has been studied by scholars in many scenarios [5,6,29,32,33,34,35]. For instance, in [6], an incentive mechanism was formulated to motivate femtocell users to act as relays for MUEs. The authors of [29] proposed an incentive framework to analyze the interactions among players. In [33], a quality of service (QoS)-based incentive mechanism was proposed to realize the efficient mobile data offloading. The researchers of [34,35] studied the incentive mechanism for cached-enabled SC sharing problem and device-to-device (D2D) content sharing and proactive caching problem, respectively.

There were also many incentive mechanisms designed to solve the traffic offloading problem in HetNets [8,9,10]. Specifically, the researchers of [9] classified incentives into five categories and investigated the offloading problem in terms of each incentive. In [8], the MBS allocated part of the subchannels to spur the femto access point to serve MUEs. The authors of [10] proposed a utility-aware refunding framework to encourage femto holders (FHs) to share resources. It is notable that, in existing studies, the reward each SC obtains is associated with the amount of resource it shares, usually the more resources, the more rewards. Typically, in [8], the more MUEs an SC serves, the more subchannels it will be given by the MBS. In [10], the more time an SC serves MUEs, the more refunding it receives. They followed this rule under the assumption that the more resources, the more sacrifices. However, it does not make sense all the time since few resources can provide a large contribution and vice versa. Therefore, we define the enhancement promotion each SC brings as the basis of the reward division to make the division method more reasonable and effective.

The Stackelberg game model has been widely used to solve problems in wireless networks [11,12,13,14,15,16,17,18,19,20,21,22,23]. Specifically, the interference mitigation problem was studied in [11,12], the spectrum sharing problem was studied in [13,14,15], the power control problem was studied in [16,17,18,19], the resource allocation problem was studied in [20] and the anti-jamming problem was studied in [21,22,23]. Different from other game models, e.g., mean-field game [36], potential game [37] and cooperative game [38,39], players in a Stackelberg game have different superiority and are classified into leaders and followers, respectively. Leaders are superior and can predict the responses of followers before making a decision. In a HetNet, the MBS, which is usually deployed by the operator, tends to be superior to SCs, which are usually deployed by individuals. Therefore, we formulate a Stackelberg game, where the MBS is the leader and SCs are followers, to solve the traffic offloading problem.

Note that some researchers have already used the Stackelberg game model to solve the traffic offloading problem in HetNets [7,10,32]. However, most of them did not consider the heterogeneous importance among the transmitted entities such as users or data. For example, users in [7] were equally important with different interest in QoS. Transmission data in [32] had no differences in superiority. The authors of [10] restricted the maximum number of offloaded MUEs to guarantee the QoS of SUEs and made no further distinction during the analysis. In fact, for a specific SC, its dedicated SUEs should be more important than offloaded MUEs and should be paid more attention. Therefore, we let SCs care more about the earning from SUEs and define the reward each SC obtains as the sum of two different weighted parts, where the one from SUEs weighs more.

The most related work is [10]. The main difference is that the location is highlighted as an important factor in this paper and it brings the following distinctions:

- In [10], it was possible for SCs to share as many resources as they can to obtain more rewards. However, the MUEs who are located near the MBS can already receive good service and need to be offloaded, while those who are located at the boundary of SCs can not receive better service even if offloaded. Therefore, the location has a great impact on the traffic offloading process.
- In [10], the amount of resources shared is the basis of the obtained reward. However, since SCs are different far away from the MBS, the same amount of resources they share will bring different enhancement for the macro network. Therefore, the reward division method proposed in [10] was not quite reasonable nor effective and we propose an approach based on the performance promotion brought to the macro network instead of the sharing of resources.

## 3. System Models and Problem Formulation

### 3.1. System Model

Consider a HetNet composed of one MBS, *S* overlaid SCs and *M* MUEs. Denote the set of SCs as $S=\left\{1,2,\cdots S\right\}$ and the set of MUEs as $M=\left\{1,2,\cdots M\right\}$. In order to enhance the spectrum utilization, the MBS and SBSs share the spectrum in the HetNet, which results in the inter-tier inference. SBSs operate on orthogonal frequencies to avoid interfering with each other. For relieving the heavy traffic of the macro network, SBSs employ a hybrid access mechanism, so that they not only serve their dedicated SUEs, but also can help serve MUEs in their transmission ranges. Denote the set of dedicated SUEs and MUEs located in SC *i* as $D_{i}=\left\{1,2,\cdots ,D_{i}\right\}$ and $M_{i}=\left\{1,2,\cdots ,M_{i}\right\}$. Note that, (in this article, each MUE is supposed to lie in no more than one SC). Downlink transmission is considered and uplink transmission can be analyzed similarly.

The rate of MUE *m* served by the MBS is denoted as

(1) $$R_{0}^{m}=\log\left(1+\frac{P_{0}\cdot d_{0,m}^{-\theta }}{\sum_{i\in S}P_{i}\cdot d_{i,m}^{-\theta }+N_{0}}\right),$$

where $P_{0}$ and $P_{i}$ are the transmission power of the MBS and SBS *i*, respectively. Meanwhile, $d_{0,m}$ and $d_{i,m}$ are distances between the MBS and MUE *m* as well as SBS *i* and MUE *m*, respectively. The parameter θ is the path loss exponent and $N_{0}$ is the background noise.

The rate of user *j* served by SC *i* is denoted as

(2) $$R_{i}^{j}=\log\left(1+\frac{P_{i}\cdot d_{i,j}^{-\theta }}{P_{0}\cdot d_{0,j}^{-\theta }+N_{0}}\right),$$

where $d_{i,j}$ and $d_{0,j}^{}$ are distances between SBS *i* and user *j* as well as the MBS and user *j*, respectively. Note that, if a MUE is offloaded to SC *i*, the rate of it should be calculated by Equation (2) as well, since the signal from the MBS it receives should be treated as the interference at this time. Meanwhile, the location of the SC each MUE lies in turns out to be an important factor influencing the rate, since the farther the SC is, the less interference MUEs suffer.

**Figure 1:**
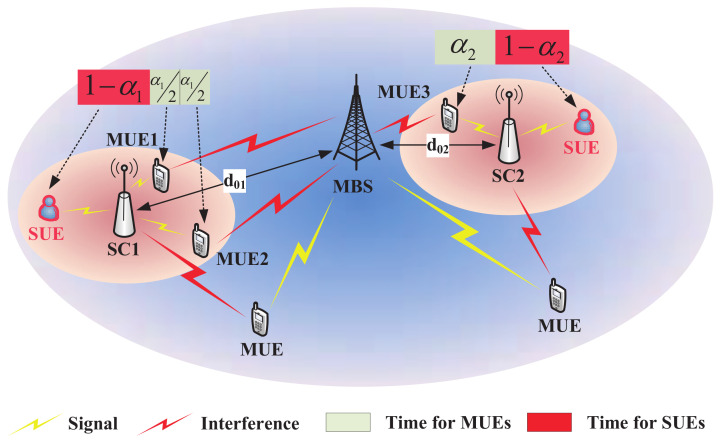
A demonstration of the system model.

Figure 1 is a brief demonstration of the system model, where MUE 1 and 2 are offloaded to SC 1 and MUE 3 is offloaded to SC 2. Compared with SC 2, SC 1 is far away from the MBS. Therefore, MUE 1 and 2 receive less interference from the MBS.

Consider a classical round robin scheduling method [40] where users served by the same base station can be served with equal probability. That is to say, when all *M* MUEs are served by the MBS, each of them can be served for a fraction of time 1/M. Supposing that SC *i* opens a fraction of time $\alpha_{i}\in \left[0,1\right]$ serving MUEs in its transmission range, MUE $m\in M_{i}$ can be served for a fraction of time $\alpha_{i}^{m}=\alpha_{i}/M_{i}$. The fraction of time reserved for its dedicated SUEs is $1-\alpha_{i}$ and SUE $s\in D_{i}$ can be served for a fraction of time $\alpha_{i}^{s}=\left(1-\alpha_{i}\right)/D_{i}$. For example, in Figure 1, since SC 1 serves both MUE 1 and 2, each of them can be served for a fraction of time $\frac{\alpha_{1}}{2}$.

The aggregate rate of MUEs located in SC *i*’s transmission range served by the MBS and that of MUEs and SUEs served by SC *i* are respectively denoted as

(3) $$R_{0i}^{MUE}=\frac{1}{M}\sum_{m\in M_{i}}R_{0}^{m},$$

(4) $$R_{i}^{MUE}=\sum_{m\in M_{i}}\alpha_{i}^{m}R_{i}^{m}=\frac{\alpha_{i}}{M_{i}}\sum_{m\in M_{i}}R_{i}^{m},$$

and

(5) $$R_{i}^{SUE}=\sum_{s\in D_{i}}\alpha_{i}^{s}R_{i}^{s}=\frac{1-\alpha_{i}}{D_{i}}\sum_{s\in D_{i}}R_{i}^{s}.$$

Denote the average rate of MUEs and SUEs served by SC *i* as $\bar{R_{i}^{m}}=\frac{\sum_{m\in M_{i}}R_{i}^{m}}{M_{i}}$ and $\bar{R_{i}^{s}}=\frac{\sum_{s\in D_{i}}R_{i}^{s}}{D_{i}}$. Then, Equations (4) and (5) can be reformulated as

(6) $$R_{i}^{MUE}=\alpha_{i}\bar{R_{i}^{m}}$$

and

(7) $$R_{i}^{SUE}=\left(1-\alpha_{i}\right)\bar{R_{i}^{s}}.$$

### 3.2. Problem Formulation

Although SBSs employ hybrid access mechanism and can serve MUEs in their transmission ranges, they need to serve their dedicated users and are selfish most of the time, which means that they will not accept MUEs willingly without any benefit. Therefore, the MBS has to provide some reward to motivate them be helpful. Denote the total reward the MBS offers as *q* and the reward SC *i* obtains as $q_{i}$, $\sum_{i\in S}q_{i}=q$.

An appropriate way to divide the reward among SCs is in proportion with their contribution. Different from most existing research where only the opening resource was taken as the contribution [5,10,29,32], we suppose that the performance promotion SCs bring for the macro network is more meaningful and practical. Therefore, in this article, the promotion of the aggregate rate of MUEs, i.e., the rate difference after and before MUEs are offloaded to SCs, is the basis of the reward division. Denote the promotion SC *i* brings as

(8) $${\tilde{R}}_{i}=R_{i}^{MUE}-R_{0i}^{MUE}.$$

The reward SC *i* obtains is in the ratio of the promotion it brings to the sum of the promotion all SCs bring. Mathematically,

(9) $$q_{i}=q\cdot \frac{{\tilde{R}}_{i}\cdot \varepsilon \left({\tilde{R}}_{i}\right)}{\sum_{j=1}^{S}\left({\tilde{R}}_{j}\cdot \varepsilon \left({\tilde{R}}_{j}\right)\right)},$$

where $\varepsilon \left({\tilde{R}}_{i}\right)=\left\{\begin{array}{c} 1,{\tilde{R}}_{i}>0\\ 0,{\tilde{R}}_{i}\leq 0 \end{array}\right.$ is an indicator function to restrict an SC obtain no reward if it brings no promotion.

The earning of each SC is composed of two parts that relatively come from its dedicated SUEs and the MBS, i.e., $q_{i}$. Denote $\omega_{s}$ as the revenue per unit rate given by SUEs. Note that, for a specific SC, its dedicated SUEs should be more important than MUEs in its transmission range. That is to say, the two parts of earning should not have the same importance. Traditionally, the part from its dedicated SUEs is more significant than that from the MBS. Therefore, denote the relative importance MUEs as $\chi_{i},\chi_{i}\in \left(0,1\right)$. Then, the earning SC *i* obtains can be defined as

(10) $$Q_{i}=\omega_{s}R_{i}^{SUE}+\chi_{i}q_{i},$$

where $R_{i}^{SUE}$ is the aggregate rate of SC *i*s’ dedicated SUEs defined in Equation (5).

Since each SC is selfish and rational, it only aims at maximizing its own earning, and the time opening problem can be formulated as

(11) $$P1:\;\alpha_{i}=\arg\max Q_{i},\forall i\in S.$$

The earning of the MBS is composed of two parts as well: the revenue from MUEs and the reward given to SCs, i.e., *q*. Motivated by [10], we introduce the churn rate to depict how many MUEs leave the macro network. Denote the churn rate as $c=\frac{1}{1+e^{-a(b-\lambda )}}$, where *a* is the MUE’s sensitivity to QoS enhancement, *b* is the reserved demand of MUEs and λ is the achievable rate of the macro network. Specifically, $\lambda =\lambda_{0}+\sum_{i=1}^{S}R_{i}^{MUE}$, where $\lambda_{0}$ is the capacity of the MBS and $R_{i}^{MUE}$ is the aggregate rate of MUEs served by SC *i* defined in Equation (4). Denote $\omega_{m}$ as the revenue when *c* drops by one percent. Then, the earning the MBS obtains can be defined as

(12) $$Q_{0}=\omega_{m}\left(1-c\right)-q.$$

Since the MBS aims at maximizing its own earning as well, the reward giving problem can be formulated as

(13) $$P2:\;q=\arg\max Q_{0}.$$

## 4. Stackelberg Game Model and Analysis

Considering different superiority of the MBS and SBSs in the HetNet, we formulate the proposed problem as a Stackelberg game, in which the MBS acts as the leader and SBSs act as followers. Since the leader is superior to followers, it has more information and can speculate their responses before making a decision.

**Definition** **1.** ***(Stackelberg equilibrium [12])** In a Stackelberg game, the strategy profile $\left(q^{*},\alpha^{*}\right)$ is an SE if the leader reaches the maximal utility at $q^{*}$ and $\alpha^{*}=\left(\alpha_{1}^{*},\alpha_{2}^{*},\cdots ,\alpha_{S}^{*}\right)$ is the best response profile of followers. Mathematically,*

(14) $$u_{0}\left(q^{*},\alpha^{*}\right)\geq u_{0}\left(q,\alpha^{*}\right),$$

(15) $$u_{i}\left(q^{*},\alpha_{i}^{*},\alpha_{-i}^{*}\right)\geq u_{i}\left(q^{*},\alpha_{i}^{},\alpha_{-i}^{*}\right),\forall i\in S,$$

*where $\alpha_{i}$ and $\alpha_{-i}$ are the strategies of player i and all players except player i, respectively.*

**Figure 2:**
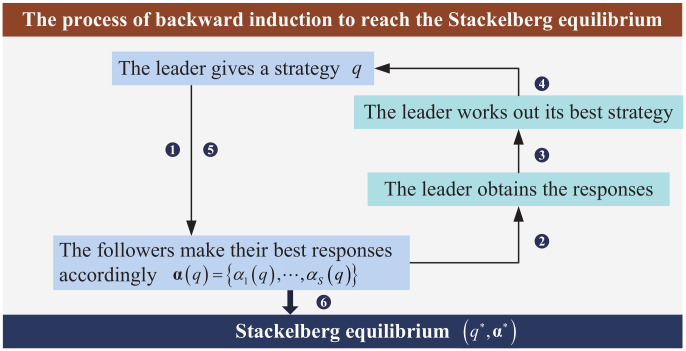
The process of the backward induction.

Since the SE can be obtained via reaching its subgame NE [12], we use the backward induction [8,16,32] to find the NE for the lower level subgame first and then the optimal strategy for the leader. The process of the method is shown in Figure 2.

### 4.1. The Lower Level Subgame of SCs

We first analyze the lower level subgame and assume the reward given by the MBS *q* is known. Each SC aims at maximizing its own earning and their strategies are coupled with each others. Therefore, the lower level subgame is a non-cooperative one and each player (SC) tries to find the best strategy given others’ strategies. The lower level subgame can be formally modeled as

(16) $$G_{l}=\left[S,{\left\{A_{i}\right\}}_{i\in S},{\left\{u_{i}\right\}}_{i\in S}\right],$$

where $S=\left\{1,2,\cdots S\right\}$ is the set of players, $A_{i}$ is the strategy space of player *i* and $u_{i}$ is the utility function of player *i*. Since the strategies of all players are coupled, the utility function of player *i* can be expressed as $u_{i}\left(\alpha_{i},\alpha_{-i}\right)$. Since each player pursues higher earning, define the utility function of player *i* as

(17) $$u_{i}=\omega_{s}R_{i}^{SUE}+\chi_{i}q_{i},$$

which is exactly the earning it obtains defined in (10). Therefore, the lower level subgame can be expressed as:

(18) $$G:\max_{\alpha_{i}\in A_{i}}u_{i}\left(\alpha_{i},\alpha_{-i}\right),\forall i\in S.$$

**Definition** **2.** ***(Nash equilibrium [41])** Only if no player can achieve better utility by unilaterally changing its action can the strategy profile $\alpha^{*}=\left(\alpha_{1}^{*},\alpha_{2}^{*},\cdots ,\alpha_{S}^{*}\right)$ be defined as an NE. Mathematically,*

(19) $$u_{i}\left(\alpha_{i}^{*},\alpha_{-i}^{*}\right)\geq u_{i}\left(\alpha_{i},\alpha_{-i}^{*}\right),\forall i\in S,\alpha_{i}\subseteq A_{i},\alpha_{i}^{*}\neq \alpha_{i}.$$

**Remark** **1.** Given the reward provided by the MBS q and the strategies of others $\alpha_{-i}$, the best response of player i is:

***if***

(20) $$F\left(\alpha_{-i}\right)<\frac{\chi_{i}q\bar{R_{i}^{m}}}{\omega_{s}\bar{R_{i}^{s}}},$$

(21) $$\alpha_{i}^{*}=\frac{\sqrt{\frac{\chi_{i}q\bar{R_{i}^{m}}F\left(\alpha_{-i}\right)}{\omega_{s}\bar{R_{i}^{s}}}}-F\left(\alpha_{-i}\right)+R_{0i}^{MUE}}{\bar{R_{i}^{m}}},$$

***else**, $\alpha_{i}^{*}=0$, where*

(22) $$F\left(\alpha_{-i}\right)=\sum_{k\in \left\{S\backslash i\right\}}f\left(\alpha_{k}\right),$$

(23) $$f\left(\alpha_{j}\right)={\tilde{R}}_{j}\cdot \varepsilon \left({\tilde{R}}_{j}\right),\forall j\in S,$$

*and A\C means C is excluded from A.*

**Proof.**  The proof follows a similar methodology in [10] and is omitted here. ☐

**Proposition** **1.** *The lower level subgame G has as a unique NE and the strategy of each player is given by Equation (27). In addition, the NE is the optimum of* **P1***.*

**Proof.**  According to Definition 2, at the NE point, each player selects its best strategy and has no incentive to deviate. Therefore, the best response of player *i* can be expressed as:

**if**

(24) $$F\left(\alpha_{-i}^{*}\right)<\frac{\chi_{i}q\bar{R_{i}^{m}}}{\omega_{s}\bar{R_{i}^{s}}},$$

(25) $$\alpha_{i}^{*}=\frac{\sqrt{\frac{\chi_{i}q\bar{R_{i}^{m}}F\left(\alpha_{-i}^{*}\right)}{\omega_{s}\bar{R_{i}^{s}}}}-F\left(\alpha_{-i}^{*}\right)+R_{0i}^{MUE}}{\bar{R_{i}^{m}}},$$

**else**, $\alpha_{i}^{*}=0$.

After calculation and deduction, the following expression of $\alpha_{i}^{*},\forall i\in S$, can be given.

**if**

(26) $$\frac{\bar{R_{i}^{s}}}{\chi_{i}\bar{R_{i}^{m}}}<\frac{\sum_{j=1}^{S}\frac{\bar{R_{j}^{s}}}{\chi_{j}\bar{R_{j}^{m}}}}{S-1}\;\;\mathrm{and}\;\;q>0,$$

(27) $$\alpha_{i}^{*}=\frac{R_{0i}^{MUE}}{\bar{R_{i}^{m}}}+\frac{\left(S-1\right)\left[\sum_{j=1}^{S}\frac{\bar{R_{j}^{s}}}{\chi_{j}\bar{R_{j}^{m}}}-\left(S-1\right)\frac{\bar{R_{i}^{s}}}{\chi_{i}\bar{R_{i}^{m}}}\right]}{\omega_{s}\bar{R_{i}^{m}}{\left(\sum_{j=1}^{S}\frac{\bar{R_{j}^{s}}}{\chi_{j}\bar{R_{j}^{m}}}\right)}^{2}}q,$$

**else**, $\alpha_{i}^{*}=0.$

According to [42], since $\alpha_{i}$ is nonempty, convex and compact and $u_{i}$ is continuous and quasi-concave in $\alpha_{i}$, there exists an NE in the lower level subgame G. Meanwhile, the expression of $\alpha_{i}^{*}$ is unique as shown in Equation (27).

Since $\alpha_{i}^{*}$ maximizes $u_{i}$, which is the same as $Q_{i},\forall i\in S$, combining Definition 2, the NE optimizes **P1** as well. This ends the proof. ☐

### 4.2. The Best Strategy of the MBS

In a Stackelberg game, the leader is superior and can predict the best response of followers before making a decision. Therefore, the utility function of the leader can be formulated as

(28) $$u_{0}=\omega_{m}\frac{1}{1+e^{-a\left(\lambda^{*}-b\right)}}-q,$$

where $\lambda^{*}=\lambda_{{}^{0}}^{}+\sum_{i=1}^{S}\alpha_{i}^{*}\bar{R_{i}^{m}}$ and other parameters are defined in Equation (12). Note that, since $\alpha_{i}^{*}$ is obtained under a given *q*, $\lambda^{*}$ is a function of *q*.

**Proposition** **2.** *When*

(29) $$\lambda_{0}>b,$$

(30) $$y>\frac{1}{2}\left(e^{a(b-\lambda_{0}-\sum_{i=1}^{S}x_{i}\bar{R_{i}^{m}})}+e^{-a(b-\lambda_{0}-\sum_{i=1}^{S}x_{i}\bar{R_{i}^{m}})}\right),$$

*the best strategy of the MBS is*

(31) $$q^{*}=\frac{1}{\sum_{i=1}^{S}\rho_{i}\bar{R_{i}^{m}}}\left(b-\frac{\ln\left(y-\sqrt{y^{2}-1}\right)}{a}-\sum_{i=1}^{S}x_{i}\bar{R_{i}^{m}}-\lambda_{0}\right),$$

*where*

(32) $$y=\frac{a\omega_{m}}{2}\sum_{i=1}^{S}\rho_{i}\bar{R_{i}^{m}}-1,$$

(33) $$\rho_{i}=\left\{\begin{array}{c} \frac{\left(S-1\right)\left[\sum_{j=1}^{S}\frac{\bar{R_{j}^{s}}}{\chi_{j}\bar{R_{j}^{m}}}-\left(S-1\right)\frac{\bar{R_{i}^{s}}}{\chi_{i}\bar{R_{i}^{m}}}\right]}{\omega_{s}\bar{R_{i}^{m}}{\left(\sum_{j=1}^{S}\frac{\bar{R_{j}^{s}}}{\chi_{j}\bar{R_{j}^{m}}}\right)}^{2}},\frac{\bar{R_{i}^{s}}}{\chi_{i}\bar{R_{i}^{m}}}<\frac{\sum_{j=1}^{S}\frac{\bar{R_{j}^{s}}}{\chi_{j}\bar{R_{j}^{m}}}}{S-1}\\ 0,\;\;\;\;\;\;\;\;\;\;\;\;\;\;\;\;\;\;\;\;\;\;\;\;\;\;\;\;\;\;\;\;\;\mathrm{else} \end{array}\right.,$$

(34) $$x_{i}=\left\{\begin{array}{c} \frac{R_{0i}^{MUE}}{\bar{R_{i}^{m}}},\frac{\bar{R_{i}^{s}}}{\chi_{i}\bar{R_{i}^{m}}}<\frac{\sum_{j=1}^{S}\frac{\bar{R_{j}^{s}}}{\chi_{j}\bar{R_{j}^{m}}}}{S-1}\\ 0,\;\;\;\;\;\;\;\;\;\;\;\;\mathrm{else} \end{array}\right..$$

**Proof.**  The following proof follows similar methodology in [10].

The first and second derivatives of $u_{0}$ with respect to *q* are as follows:

(35) $$\frac{\partial u_{0}}{\partial q}=-1+a\omega_{m}\sum_{i=1}^{S}\rho_{i}\bar{R_{i}^{m}}\frac{e^{-a(\lambda^{*}-b)}}{{\left[1+e^{-a(\lambda^{*}-b)}\right]}^{2}},$$

(36) $$\frac{\partial^{2}u_{0}}{\partial q_{}^{2}}=\omega_{m}{\left(a\sum_{i=1}^{K}\rho_{i}\bar{R_{i}^{m}}\right)}^{2}\frac{e^{-a(\lambda^{*}-b)}\left(e^{-a(\lambda^{*}-b)}-1\right)}{{\left(1+e^{-a(\lambda^{*}-b)}\right)}^{3}}.$$

When Equation (29) is satisfied, the second derivative of $u_{0}$ is always less than zero, i.e., $\frac{\partial^{2}u_{0}}{\partial \alpha_{i}^{2}}<0$. That is to say, $u_{0}$ is concave in *q* and has a maximum. Making the first derivative of $u_{0}$ equal zero, i.e., $\frac{\partial u_{0}}{\partial q}=0$, $u_{0}$ will reach its maximal value when *q* is set as Equation (31). When Equation (30) is satisfied, $q^{*}>0$ holds. Therefore, the best strategy of the MBS is proved. ☐

**Proposition** **3.** The proposed Stackelberg game has a unique SE.

**Proof.**  Based on Proposition 1 and 2, it can be known that the lower level subgame has a unique NE and the best strategy of the MBS is unique, i.e., the strategy profile $\left(q^{*},\alpha^{*}\right)$ is unique. Therefore, according to Definition 1, $\left(q^{*},\alpha^{*}\right)$ is the unique SE of the Stackelberg game, where $\alpha_{i}^{*},\forall i\in S$ is defined as Equation (27) and $q^{*}$ is defined as Equation (31). ☐

### 4.3. The Process of Traffic Offloading

Through the analysis above, it can be known that the unique SE of the proposed Stackelberg game can be achieved when the MBS and all SCs choose their optimal strategies. In this subsection, we explain the process of the proposed traffic offloading problem.

- The MBS has superiority and can predict the responses of SCs. It first checks whether Equations (29) and (30) are satisfied. If so, it announces the optimal reward according to Equation (31). Otherwise, it does not provide any reward.
- SCs move after the MBS and make the decisions. Once the reward provided by the MBS is announced, SCs check whether Equation (26) is satisfied. If so, they set the opening time ratio according to Equation (27) in a distributed way. Otherwise, they do not provide any service for MUEs.

### 4.4. Complexity Analysis

To better understand the proposed method, we analyze the complexity cost in this subsection, which refers to [43].

- Before the leader takes action, it requires information from all followers to make the judgement. The complexity of this process is $O\left(S\right)$, where *S* is the number of followers.
- When the leader takes action, since it already has the information from all followers, the complexity of this process is $O\left(C_{1}\right)$, where $C_{1}$ is a small constant.
- After the strategy of the leader is given, the followers require information from all other followers to make a judgement. The complexity of this process is $S\cdot O\left(S\right)$.
- When the leader takes action, since they already have the information from other followers, the complexity of this process is $O\left(C_{2}\right)$, where $C_{2}$ is a small constant.

Therefore, the total complexity of the used method can be expressed as

(37) $$C=O\left(S\right)+O\left(C_{1}\right)+O\left(S\right)+O\left(C_{2}\right).$$

It can be seen that the complexity scales linearly with the number of SCs. The complexity is low when there are not many SCs and it rises slowly with the growing number of SCs.

## 5. Simulation and Numerical Results and Discussion

In this section, the effectiveness and the superiority of the proposed method are verified. Some basic network parameters are set as follows. The HetNet is a 200 m × 200 m one where the MBS is at the center. The transmission powers of the MBS and each SBS are 46 dBm and 23 dBm, respectively. The transmission range of each SC is 20 m. The path loss exponent is θ=3 and the background noise is $N_{0}=-120$ dBm[18,19]. The unit revenue gained from MUEs and SUEs, i.e., $\omega_{m}$ and $\omega_{s}$, are 5 and 0.1, respectively. The relative importance of MUEs is χ=0.7. The user’s sensitivity to QoS increment is a=2. The reserved traffic demand of MUEs is b=0.2. The original traffic capacity of the MBS is $\lambda_{0}=0.21$ [10]. Note that the result in Section 5.1 is obtained under a given network while the results in Section 5.2, Section 5.3, Section 5.4 and Section 5.5 are obtained by averaging 1000 times of simulation results under randomly generated networks, where the locations of SCs and users change each time.

### 5.1. The Influence of Location

**Figure 3:**
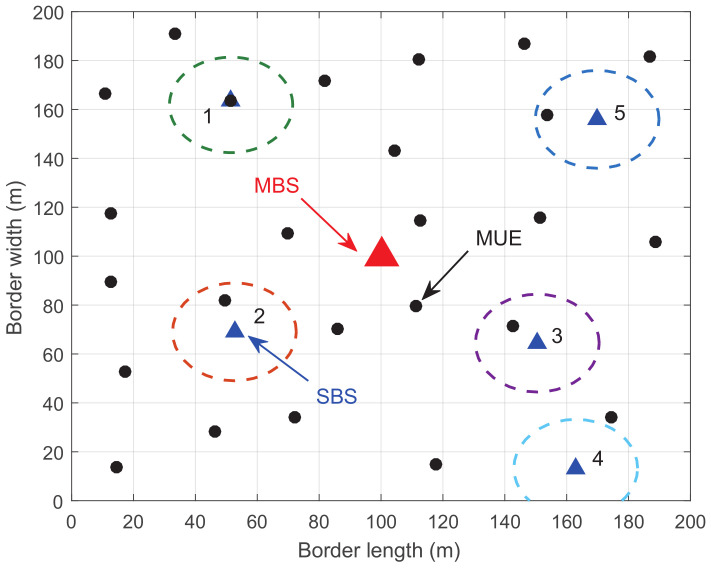
The topology of the simulated network.

In order to exhibit the influence of location of SCs, we first make simulations in a given network shown in Figure 3. There are five SCs and 25 MUEs in the network. Suppose each SC possesses one SUE that is not shown in the figure. The distances between each SC and the MBS are heterogeneous. Obviously, SC 2 is the nearest one to the MBS while SC 4 is the farthest one. The distances between the MUEs in each SC and the MBS are heterogeneous as well. Generally, MUEs in SC 2 can receive better service than those in SC 5 from the MBS.

**Figure 4:**
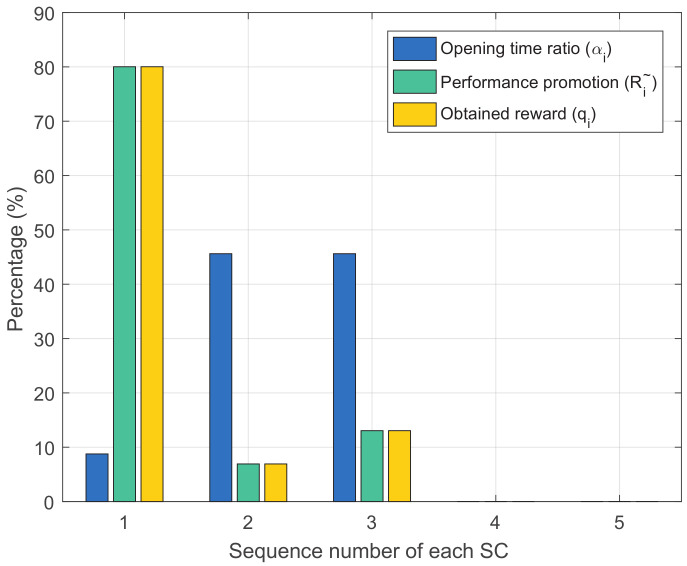
The opening time ratio, the performance promotion and the reward of different SCs.

Figure 4 presents the relationship between the opening time ratio, the performance promotion brought to the macro network and the reward of each SC via the proposed method. It can be seen that the opening time ratio and the reward is not in a positive proportional relationship. Specifically, SC 2 and 3 provide more time ratio than SC 1 but receive less rewards. This is because the former two SCs are closer to the MBS. Therefore, MUEs lying in them receive better service before offloading than those lying in SC 1. That is to say, the promotion SC 2 and 3 bring to the macro network is less evident than SC 1 and will consequently obtain less rewards. Note that, if an SC can not bring better service to the MUE, it does not serve it, e.g., SC 5. Meanwhile, if an SC does not serve any MUE, it obtains no reward, e.g., SC 4 and SC 5.

### 5.2. The Influence of Load

**Figure 5:**
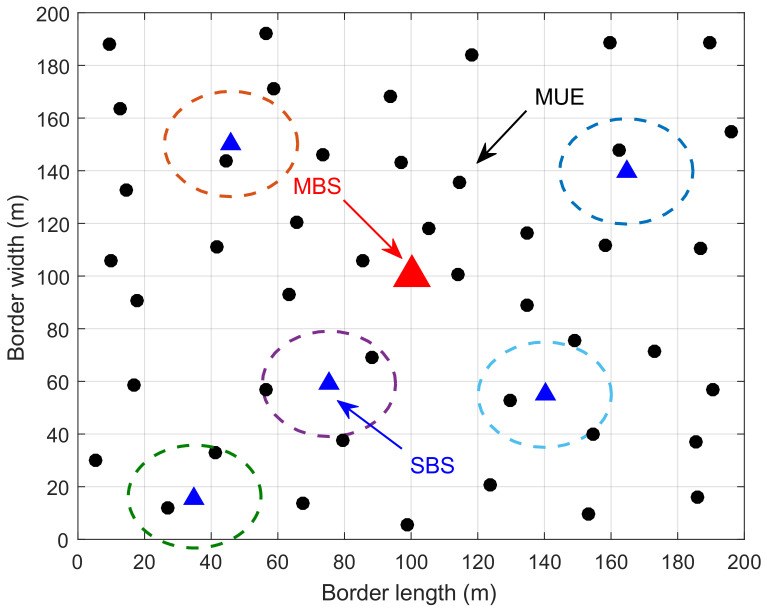
The topology of a dense network with 45 MUEs.

**Figure 6:**
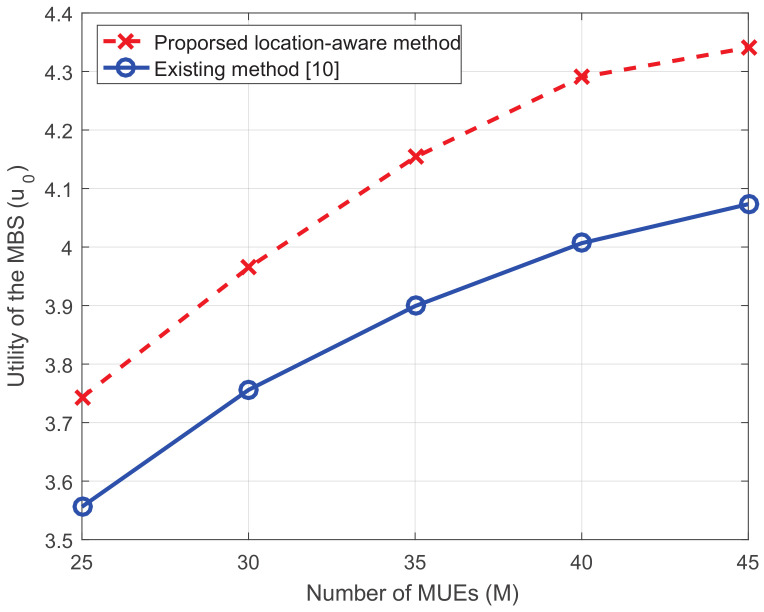
The utility of the MBS versus the number of MUEs.

In order to show the influence of the load of the macro network, we change the number of MUEs from 25 to 45, where the density of the most intensive network is shown in Figure 5. It can be seen from Figure 6 that the proposed method is always superior to the existing one. With the heavier weight of the load, the utility of the MBS increases. This is because more MUEs lie in SCs and more MUEs can receive better service after offloading.

### 5.3. The Influence of the Number of SCs

**Figure 7:**
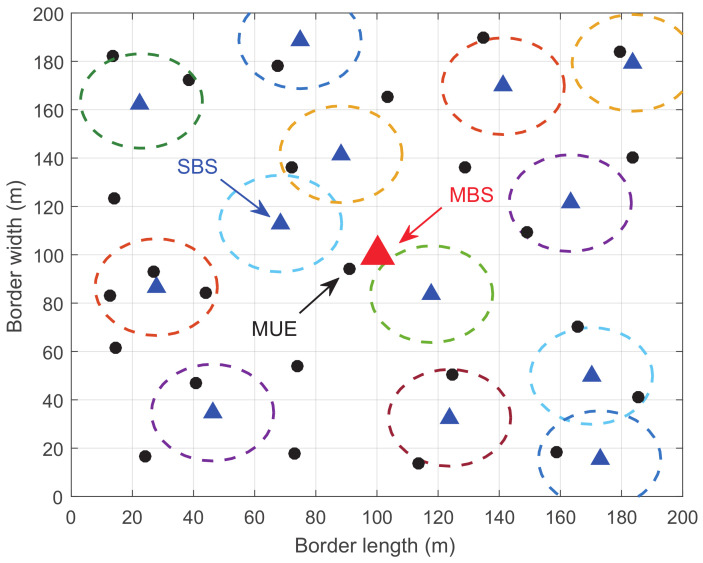
The topology of a network with 13 SCs.

**Figure 8:**
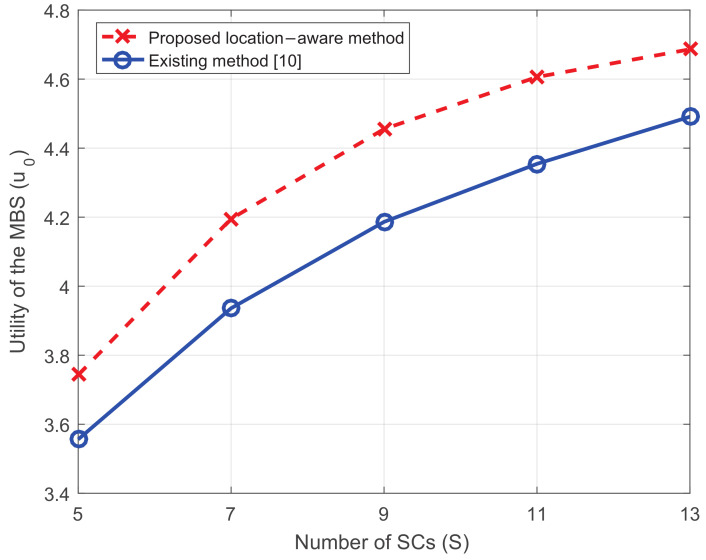
The utility of the MBS versus the number of SCs.

In order to show the influence of the number of SCs, we change it from 5 to 13, where the density of the network with the most SCs can be seen in Figure 7. Figure 8 shows the influence of the number of SCs. It can be seen that, when the number of SCs turn larger, the utility of the MBS increases.

This can be explained mathematically by (31) where the reward the MBS offers is in a negative relationship with the number of SCs, which will result in higher utility. Practically, when there are more SCs in the network, they will be more willing to serve MUEs since the competition for the reward turns more severe. In this way, the MBS will reduce the reward naturally.

### 5.4. The Influence of the Sensitivity of MUEs

**Figure 9:**
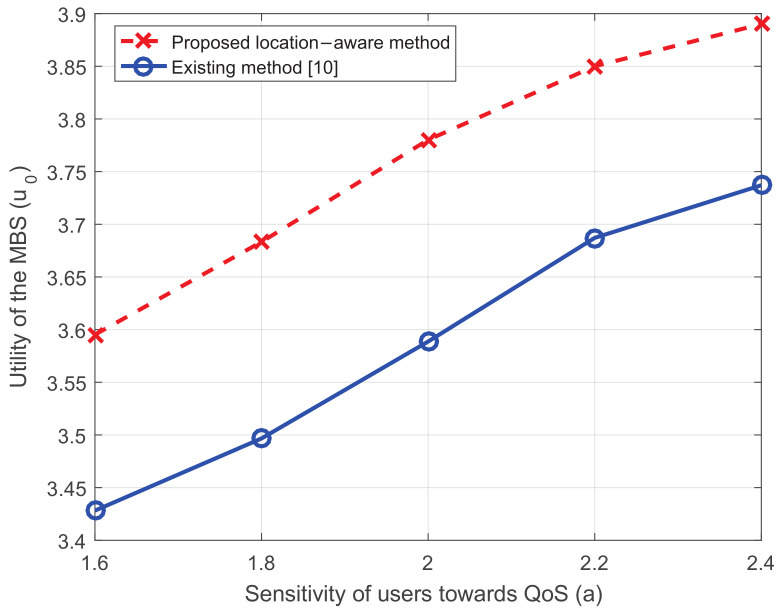
The utility of the MBS versus the sensibility of users towards QoS.

Figure 9 shows the influence of the sensitivity of MUEs towards QoS. Note that the more sensitive MUEs are, i.e., the larger *a* is, the more dramatically the churn rate *c* drops when QoS upgrades [10]. It can be seen that, when MUEs turn more sensitive, the utility of the MBS increases. This is because more MUEs are willing to stay with the macro network as they can experience better QoS.

### 5.5. The Influence of the Importance of MUEs

**Figure 10:**
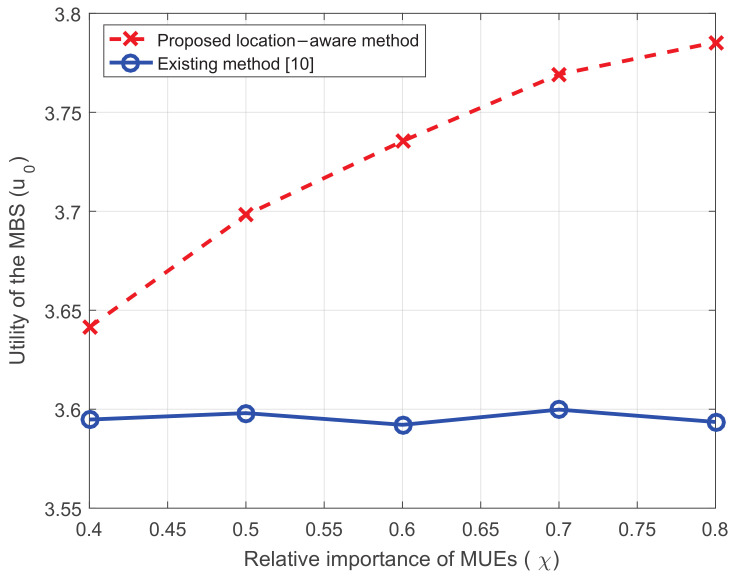
The utility of the MBS versus the relative importance of MUEs.

Figure 10 shows the influence of the relative importance of MUEs. The existing method does not consider the parameter and the utility of the MBS does not change with it. In the proposed method, when MUEs become more important, the utility of the MBS increases. This is because SCs care them more and spend more resources on them.

## 6. Conclusions

In this article, the traffic offloading problem in a heterogeneous network was investigated. In order to stimulate SCs to serve MUEs, a location-aware incentive mechanism was designed. The location of SCs was viewed as an important factor in influencing the network performance. Different from most existing work where the reward division among SCs was based on the amount of resources shared, the performance promotion each SC brought to the macro network was taken as the basis. In addition, the significance of SUEs and MUEs was distinguished instead of being treated equally by SCs. The problem was formulated as a Stackelberg game where the MBS acted as the leader and SCs were the followers. It was proven that the proposed game has a unique SE, and the SE was exactly the optimum of the proposed problem. Simulation and numerical results verified the effectiveness of the proposed method.
